# The value of endocervical curettage for diagnosis of cervical precancers or worse at colposcopy of women with atypical glandular cells cytology

**DOI:** 10.3389/fmed.2024.1476361

**Published:** 2024-12-13

**Authors:** Yusha Chen, Fanghong Wen, Jiancui Chen, Huifeng Xue, Xiangqin Zheng, Diling Pan

**Affiliations:** ^1^Cervical Disease Diagnosis and Treatment Health Center, Fujian Maternity and Child Health Hospital College of Clinical Medical for Obstetrics & Gynecology and Pediatrics, Fujian Medical University, Fuzhou, China; ^2^Department of Obstetrics, Fujian Maternity and Child Health Hospital College of Clinical Medical for Obstetrics & Gynecology and Pediatrics, Fujian Medical University, Fuzhou, China; ^3^Department of Gynecology, Fujian Maternity and Child Health Hospital College of Clinical Medical for Obstetrics & Gynecology and Pediatrics, Fujian Medical University, Fuzhou, China; ^4^Department of Pathology, Fujian Maternity and Child Health Hospital College of Clinical Medical for Obstetrics & Gynecology and Pediatrics, Fujian Medical University, Fuzhou, China

**Keywords:** cervical curettage, colposcopy, biopsy, cervical lesions, atypical glandular cells

## Abstract

**Objective:**

This study evaluates the effectiveness of endocervical curettage (ECC) in identifying additional cervical cancer and its precursors in women with atypical glandular cells (AGC) cytology.

**Methods:**

We conducted a retrospective analysis of medical records for women referred to colposcopy with AGC cytology between January 2019 and December 2023. The study included 433 women with AGC cytology who underwent both biopsy and ECC. Clinical characteristics such as demographics, clinical history, cytology, HPV status, colposcopic findings, and pathology were analyzed. Chi-square and Fisher's exact tests were applied to compare the characteristics of ECC-diagnosed cervical precancers or worse (HSIL+) and normal/low-grade squamous intraepithelial lesions (LSIL).

**Results:**

The overall detection rate of HSIL+ in this population was 19.4% (86/443), with ECC alone identifying HSIL+ in 1.3% (6/443) of cases. However, ECC showed greater utility in certain subgroups. The highest additional HSIL+ detection from ECC was observed in women with HPV 16/18 infection (7.2%) and those with AGC-FN cytology (4.4%). ECC's additional yield of HSIL+ was higher in those with normal or LSIL colposcopic impressions compared to those with HSIL+ impressions. Conversely, no additional HSIL+ cases were identified by ECC alone in women under 30 years old, those with negative high-risk HPV results, or those with type 1/2 transformation zones.

**Conclusion:**

For women with AGC cytology, ECC should be performed in patients with AGC-FN cytology, HPV 16/18 infections, type 3 transformation zones, and normal or low-grade colposcopic impressions. This approach enhances the identification of HSIL+ cases by reducing false negatives. However, for women younger than 30 years old and those with type 1/2 transformation zones, ECC offers limited benefit.

## 1 Introduction

Colposcopy is an important diagnostic procedure for identifying precancerous lesions of the cervix, offering a thorough examination to detect and assess abnormal areas. Nevertheless, the endocervical canal often remains difficult to visualize during colposcopy, which can result in missed significant lesions. Lesions of the cervical glandular epithelium can arise not only from the squamocolumnar junction but also from columnar cells situated further up the cervical canal, frequently involving the endocervical canal. Atypical glandular cells (AGC) are the most common cytological type of cervical glandular epithelial lesions, which include adenocarcinoma *in situ* (AIS) and adenocarcinoma (1).

Endocervical curettage (ECC) involves scraping cells from the endocervical canal and is used to detect abnormalities in this otherwise challenging-to-access area. The effectiveness of ECC in lowering the miss rate of colposcopy findings remains controversial. The Society of Canadian Colposcopists (2) and the American Society for Colposcopy and Cervical Pathology (ASCCP) (3) advocate for ECC in cases where atypical glandular cells are found in cytology. In contrast, the British Society for Colposcopy and Cervical Pathology (BSCCP) (4) does not support the routine use of ECC during colposcopy, even for glandular lesions.

Research has shown that ECC can effectively detect additional cervical cancer and its precursors that might be overlooked by biopsy alone in women with high-risk cytology, including atypical squamous cells, favor high-grade (ASC-H), high-grade squamous intraepithelial lesions (HSIL), and AGC (5–7). Given its rarity—typically < 1% of cervical smear results (8)—AGC has not been extensively examined as a standalone condition. In China, ECC is routinely performed when AGC is identified cytologically, providing an opportunity to evaluate its practical effectiveness.

The Cervical Disease Diagnosis and Treatment Health Center at Fujian Maternity and Child Health Hospital, the largest center for cervical diseases in Fujian province, China, has a robust data collection system that includes histopathology, cytopathology, colposcopic findings, and patient details from all colposcopy exams. This retrospective study utilizes this comprehensive dataset to assess how effectively ECC identifies additional cervical cancer and its precursors in women with AGC cytology.

## 2 Materials and methods

### 2.1 Study design

**Figure 1:**
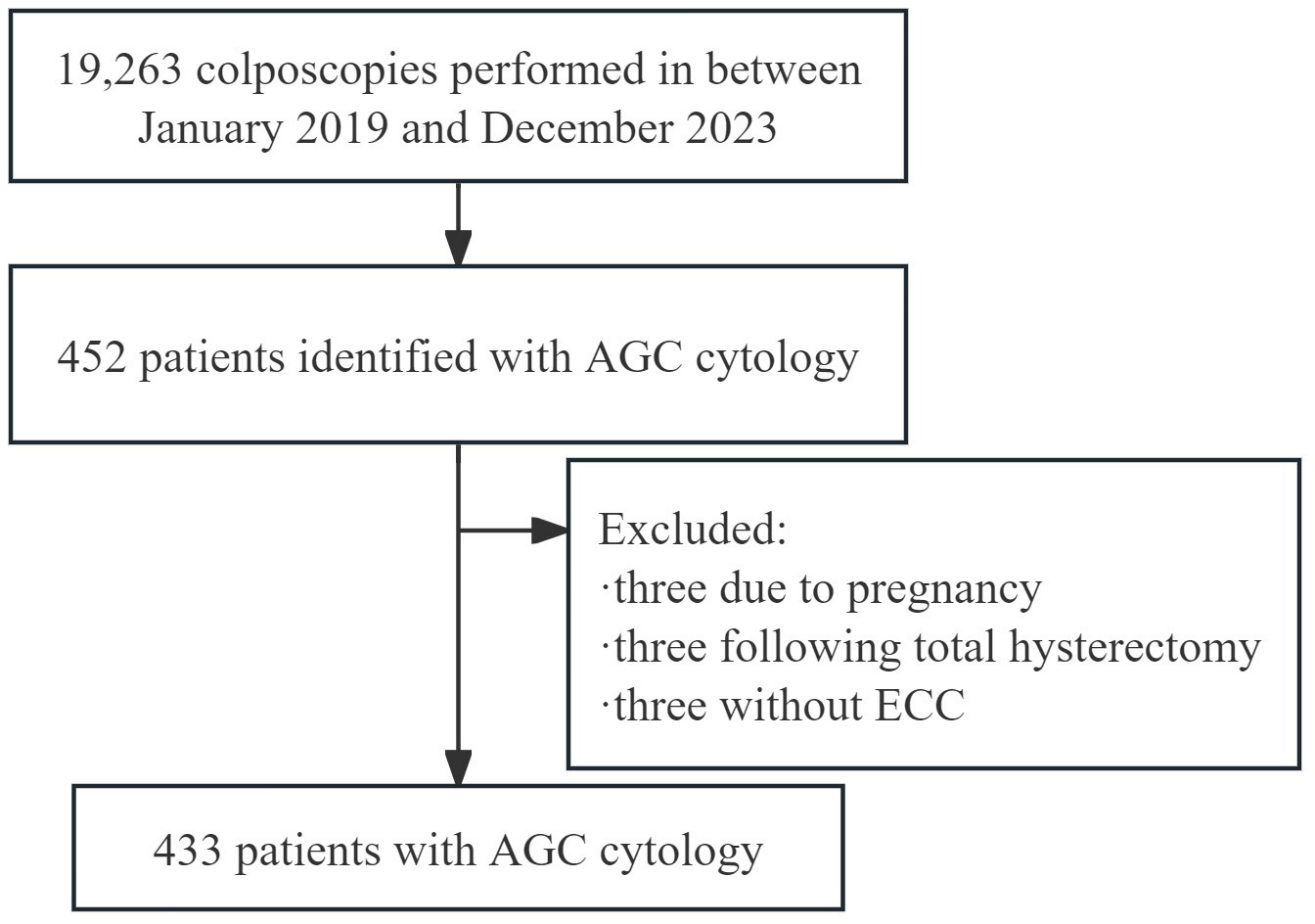
Flow chart of the study. AGC, atypical glandular cells; ECC, endocervical curettage.

Data were collected from the electronic medical records of women who underwent colposcopic examinations following abnormal cervical screening results at the Cervical Disease Diagnosis and Treatment Health Center of Fujian Maternity and Child Health Hospital. The patient selection process is detailed in Figure 1. Between January 2019 and December 2023, 19,263 colposcopies were performed at the center. Out of the 452 patients identified with AGC cytology, nine were excluded: three due to pregnancy, three following total hysterectomy, and three because of a closed cervical ostium that made endocervical curettage impossible. Therefore, the study included 433 patients with AGC cytology who had both biopsy and ECC. The time between cytology/HPV testing and colposcopy was always < 3 months. Collected demographic and clinical information included age, gravidity, parity, menopausal status, cytological results, HPV status, colposcopic impressions, transformation zone type, and pathological findings. This study was approved by the Ethics Committee at Fujian Maternity and Child Health Hospital, Affiliated Hospital of Fujian Medical University (2024KY014). Informed consent was waived due to the retrospective nature of the study. The findings were reported in accordance with the Strengthening the Reporting of Observational Studies in Epidemiology (STROBE) guidelines.

### 2.2 Cytology and HPV testing

Cytology was conducted using the liquid-based ThinPrep test. In this procedure, a cell brush is inserted into the external cervical canal to collect cells from the exocervix and endocervix. These cells are then smeared onto a slide and fixed. According to the Bethesda System (9), atypical glandular cells (AGC) can be categorized into AGC-NOS (not otherwise specified, including endocervical and endometrial) and AGC-FN (favor neoplastic).

A total of three HPV testing approaches were utilized: the hybrid capture 2 assay (Qiagen, Hilden, Germany), which detects the DNA of 13 high-risk oncogenic HPV types (including HPV types 16, 18, 31, 33, 35, 39, 45, 51, 52, 56, 58, 59, and 68); the polymerase chain reaction-reverse dot blot (PCR-RDB) HPV genotyping method (Yaneng^®^ Limited Corporation, Shenzhen, China), capable of identifying 18 high-risk HPV types (such as HPV 16, 18, 31, 33, 35, 39, 45, 51, 52, 53, 56, 58, 59, 66, 68, 73, 82, and 83) along with 5 low-risk types (6, 11, 42, 43, and 81); and the Aptima assay (Gen-Probe Inc., San Diego, CA), which focuses on the E6/E7 mRNA of 14 high-risk HPV types (including HPV 16, 18, 31, 33, 35, 39, 45, 51, 52, 56, 58, 59, 66, and 68). HPV status is classified as HPV 16/18, non-16/18 high-risk HPV, or negative.

### 2.3 Colposcopy/ECC and biopsy procedures

All patients with AGC-NOS (endometrial) and negative high-risk HPV results underwent a segmented curettage procedure before colposcopy, with no abnormalities detected in the histopathology. Colposcopic examinations were conducted using a dynamic spectral imaging (DSI) colposcope (Leisegang, Germany). Up to four lesion-directed biopsies were obtained from distinct areas of the epithelium that showed acetowhitening, metaplasia, or other visible abnormalities after the application of 5% acetic acid and Lugol's iodine using Tischler biopsy forceps. If fewer than four directed biopsies were collected, additional biopsies were taken from areas of normal-appearing cervical transformation zone epithelium until a total of four biopsies were achieved. If the cervix appeared entirely normal, random biopsies were performed at the squamocolumnar junction (SCJ) in the 3, 6, 9, or 12 o'clock positions, assuming the SCJ was fully visualized. When the SCJ was not completely visible, biopsies were collected from the external cervical ostium at the corresponding 3, 6, 9, or 12 o'clock positions. Following cervical biopsy, endocervical curettage (ECC) was performed using a Kevorkian curette. Results from ECC and biopsies were categorized as normal, low-grade squamous intraepithelial lesions (LSIL), HSIL, or invasive cancer based on the Lower Anogenital Squamous Terminology system (10). The diagnosis was based on the highest grade lesion identified, with HSIL+ encompassing HSIL, adenocarcinoma *in situ* (AIS), and invasive cancers, while all other findings were classified as < HSIL. The pathological evaluation of cervical biopsies and ECC samples was carried out independently by two senior pathologists who were blinded to each other's results.

### 2.4 Statistical analysis

Statistical analysis was performed using IBM SPSS Statistics version 26.0 (IBM Corp, Armonk, NY). Statistical significance was determined using two-sided tests with a threshold of *P* < 0.05. Categorical variables were expressed as frequencies and percentages. For analysis, chi-square tests were used, and Fisher's exact test was applied when appropriate.

## 3 Results

### 3.1 Study sample and data features

Table 1 provides an overview of the demographic and clinical characteristics of the study participants. The mean age of the patients was 41.4 years, with a range spanning from 20 to 77 years. Among the women, 81.7% (*n* = 362) had experienced one to three childbirths, and a majority, 86% (*n* = 381), were premenopausal. Additionally, 54.2% (*n* = 240) had a transformation zone classified as type 3. The most common cytological result was AGC-NOS, which constituted 78.5% (*n* = 348) of the cases. HPV 16/18 infections were detected in 15.6% (*n* = 69) of the participants, whereas 22.4% (*n* = 99) had infections with other high-risk HPV types. Colposcopic findings were normal in 32.7% (*n* = 145) of cases, low-grade in 51.7% (*n* = 229), high-grade in 12.2% (*n* = 54), and cervical cancer was diagnosed in 3.4% (*n* = 15). Histopathological examination of biopsies revealed HSIL+ in 18.1% (*n* = 80) of cases, while ECC histopathology identified HSIL+ in 7.9% (*n* = 35). Overall, 11.7% (*n* = 52) of the cases were diagnosed with HSIL, 3.8% (*n* = 17) with AIS, 1.4% (*n* = 6) with SCC (cervical squamous carcinoma), and 2.5% (*n* = 11) with ACC (cervical adenocarcinoma).

**Table 1:** Clinical features of the study cohort.

**Characteristics**	**Total patients (N)**	**Percentage(%)**
Age, median (range)	41.4	20–77
**Gravidity**		
0	43	9.7
1–3	293	66.1
>3	107	24.2
**Parity**		
0	72	16.3
1–3	362	81.7
>3	9	2
**Menopause**		
No	381	86
Yes	62	14
**Transformation zone type**		
1/2	203	45.8
3	240	54.2
**HPV status**		
Negative	246	55.5
Non-16/18 hrHPV	99	22.4
HPV16/18	69	15.6
Unknown or not performed	29	6.5
**Colposcopic impressions**		
Normal/benign	145	32.7
Low-grade	229	51.7
High-grade	54	12.2
Cervical cancer	15	3.4
**ECC**		
< HSIL	408	92.1
HSIL+	35	7.9
**Biopsy**		
< HSIL	363	81.9
HSIL+	80	18.1
**Cytology**		
AGC-NOS(endocervical)	11	2.5
AGC-NOS^*^	348	78.5
AGC-NOS(endometrial)	39	8.8
AGC-FN	45	10.2
**Histology**		
Normal	327	73.8
LSIL	30	6.8
HSIL	52	11.7
AIS	17	3.8
SCC	6	1.4
ACC	11	2.5

hr-HPV, high risk human papillomavirus; AGC-NOS, atypical glandular cells-not otherwise specified; AGC-FN, atypical glandular cells - favor neoplasia; AIS, endocervical adenocarcinoma *in situ*; LSIL, low-grade squamous intraepithelial lesion; HSIL, high-grade squamous intraepithelial lesion; ECC, endocervical curettage. AGC-NOS^*^: atypical glandular cells-not otherwise specified, the origin cannot be determined.

### 3.2 HSIL+ diagnostic yield by biopsy and ECC

The diagnostic yield for HSIL+ by biopsy and ECC is presented in Table 2. The overall concordance rate between histopathologic results of biopsy and ECC was 81.3% (360/443). In 17.4% (77/443) of cases, the biopsy revealed a higher grade of pathologic results than ECC. Conversely, ECC showed a higher grade of pathologic results in 1.3% (6/443) of cases, including 4 cases of HSIL, 1 case of AIS, and 1 case of ACC, with corresponding biopsy pathology results being normal. Biopsy alone detected 93% (80/86) of HSIL+ cases missed by ECC. Additionally, 7.7% (4/52) of HSIL cases, 5.9% (1/17) of AIS cases, and 9.0% (1/11) of ACC cases were missed by biopsy alone but were detected when biopsy was combined with ECC.

**Table 2:** Comparison of biopsy and ECC histopathology diagnostic results.

**Histopathology diagnosis by ECC**	**Biopsy histopathology diagnosis**												**Total**
	**Normal**	**%**	**LSIL**	**%**	**HSIL**	**%**	**AIS**	**%**	**SCC**	**%**	**ACC**	**%**	
Normal	328	74.0	25	5.6	32	7.2	10	2.3	1	0.2	5	1.1	401
LSIL	0	0.0	4	0.9	3	0.7	0	0.0	0	0.0	0	0.0	7
HSIL	4	0.9	0	0.0	13	2.9	0	0.0	0	0.0	0	0.0	17
AIS	1	0.2	0	0.0	1	0.2	5	1.1	0	0.0	1	0.2	8
SCC	0	0.0	0	0.0	0	0.0	0	0.0	3	0.7	0	0.0	3
ACC	1	0.2	0	0.0	0	0.0	0	0.0	2	0.5	4	0.9	7
Total	334	75.4	29	6.5	49	11.1	15	3.4	6	1.4	10	2.3	443

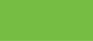
, ECC under diagnosis (*n* = 77); 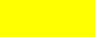, concordant diagnosis (*n* = 360); 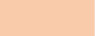, ECC over diagnosis (*n* = 6).

### 3.3 Clinical characteristics associated with HSIL+ diagnostic yield by ECC

Table 3 presents the clinical features linked to HSIL+ detection via ECC. There was no significant difference between different age groups in terms of ECC-detected normal/LSIL and HSIL+ results (*P* = 0.257). Women with AGC-FN had a higher likelihood of HSIL+ results on ECC compared to those with AGC-NOS (28.6% vs. 8.6%, *P* = 0.001). The HPV16/18 positive group exhibited the highest rate of HSIL+ detection by ECC, whereas the high-risk HPV negative group was more likely to present normal or LSIL results (*P* < 0.001). Notably, HSIL+ was more frequently detected by ECC when colposcopy impressions were normal or LSIL compared to when colposcopy impressions were HSIL+ (*P* < 0.001). All HSIL+ ECC results were found in type 3 transformation zones. Although menopausal women showed a higher propensity for HSIL+ ECC results (25.7% vs. 13.0%), this finding was not statistically significant (*P* = 0.07).

**Table 3:** Comparison of clinical characteristics between normal/LSIL and HSIL+ identified by ECC.

**Subgroups**	**Total patients (N)**	** < HSIL**	**HSIL+**	***P***
**Age (years)**				
< 30		38 (9.3%)^a^	2 (5.7%)^a^	0.257^*^
30–39		158 (38.7%)^a^	9 (25.7%)^a^	
40–49		138 (33.8%)^a^	14 (40.0%)^a^	
≥50		74 (18.1%)^a^	10 (28.6%)^a^	
**Cytology**	443			
AGC-NOS		373 (91.4%)	25 (71.4%)	0.001^*^
AGC-FN		35 (8.6%)	10 (28.6%)	
**HPV status**	414			
Negative		245 (64.5%)^a^	1 (2.9%)^b^	< 0.001
HPV16/18		45 (11.8%)^a^	24 (70.6%)^b^	
non-16/18 hr-HPV		90 (23.7%)^a^	9 (26.5%)^a^	
**Colposcopic impressions**	443			
Normal		139 (34.1)^a^	6 (17.1%)^b^	< 0.001
LSIL		220 (53.9)^a^	9 (25.7%)^b^	
HSIL+		49 (12.0)^a^	20 (57.1)^b^	
**Transformation zone type**	443			
1/2		408 (100)	16 (45.7)	< 0.001^*^
3		0	19 (54.3)	
**Menopause**	443			
No		355 (87.0)	26 (74.3)	0.07^*^
Yes		53 (13.0)	9 (25.7)	

^a, b^Different alphabet means statistically significant (*p* < 0.05). ^*^Fisher's exact test.

### 3.4 Stratification of HSIL+ diagnostic yield based solely on ECC

Table 4 presents the rates of HSIL+ detection via ECC and biopsy, organized by age group, cytology, HPV infection, colposcopic impression, transformation zone type, and menstrual status. The additional diagnostic yield of HSIL+ by ECC varied from 0.0% to 7.2% across different risk categories. The highest yield of HSIL+ was observed in women with HPV16/18 infection, reaching 7.2% (5/69). ECC identified an additional 4.4% (2/45) of HSIL+ cases in women with AGC-FN cytology. The most notable increase in detection rate from ECC was in patients aged 40–49, at 2.0% (3/152). For women with normal or LSIL colposcopic impressions, the HSIL+ diagnostic yield by ECC was 3.4% (6/374), whereas in the type 3 transformation zone group, ECC detected 2.5% (6/240) more HSIL+ cases. ECC also identified HSIL+ in 1.3% (5/381) of premenopausal women and 1.6% (1/62) of postmenopausal women, cases that were missed by biopsy alone. In contrast, ECC did not detect any additional HSIL+ cases in patients under 30 years, those with negative high-risk HPV infection, HSIL+ colposcopic impressions, or type 1/2 transformation zones.

**Table 4:** Stratified diagnostic yield of HSIL+.

**Subgroups**	**Total**	**HSIL+ yield by biopsies (*n =* 80)**	**Rate%**	**HSIL+ yield both by biopsies and ECC (*n =* 29)**	**Rate%**	**Additional HSIL+ yield ECC (*n =* 6)**	**Rate%**
**Age (years)**							
< 30	40	4	10.0	2	5.0	0	0.0
30–39	167	31	18.6	7	4.2	2	1.2
40–49	152	27	17.8	11	7.2	3	2.0
≥50	84	18	21.4	9	10.7	1	1.2
**Referral cytology grade**							
AGC-NOS	398	58	14.6	21	5.3	4	1.0
AGC-FN	45	22	48.9	8	17.8	2	4.4
**HPV status**							
Negative	246	6	2.4	1	0.4	0	0.0
HPV16/18	69	44	63.8	19	27.5	5	7.2
Non-16/18 high risk HPV	99	28	28.3	8	8.1	1	1.0
**Colposcopic impressions**							
Normal	145	8	5.5	3	2.1	3	2.1
LSIL	229	26	11.4	6	2.6	3	1.3
HSIL+	69	45	65.2	30	43.5	0	0.0
**Transformation zone type**							
1/2	203	38	18.7	9	4.4	0	0.0
3	240	42	17.5	20	8.3	6	2.5
**Menopause**							
No	381	67	17.6	21	5.5	5	1.3
Yes	62	13	21.0	8	12.9	1	1.6

## 4 Discussion

This study evaluated the effectiveness of ECC in identifying HSIL+ among women referred for colposcopy due to AGC cytology. The results revealed an overall HSIL+ detection rate of 19.4% (86/443) in this population, with ECC alone detecting HSIL+ in 1.3% (6/443) of cases. This suggests that approximately 100 women would need to endure the extended discomfort of ECC procedures to identify one additional HSIL+ case not captured by biopsy alone. Nonetheless, ECC proved more beneficial in specific subgroups. The rate of HSIL+ detection by ECC alone increased to 2.0% (8/631) among women aged 40–49 years. The highest additional HSIL+ detection from ECC was observed in women infected with HPV 16/18, at 7.2%, and in those with AGC-FN cytology, where the additional detection rate was 4.4%. ECC's additional yield of HSIL+ was higher in the group with normal or LSIL colposcopic impressions compared to the HSIL+ colposcopic impression group. Conversely, no additional HSIL+ cases were missed by biopsy alone in women under 30 years of age, those with negative high-risk HPV results, or those with type 1/2 transformation zones.

AGC represent cytological abnormalities in which glandular cells show changes but do not meet the criteria for adenocarcinoma *in situ* or invasive adenocarcinoma of the cervix uteri. These abnormalities can range from benign changes and cervical precursor lesions of glandular or squamous origins to invasive cervical cancer and other gynecological malignancies (8). This study found that 19.4% of women with AGC had cervical intraepithelial neoplasia grade two or worse, including adenocarcinoma *in situ* (CIN2+/AIS+), a figure comparable to a meta-analysis (11) reporting that 19.8% of women with AGC will have high-grade squamous intraepithelial lesions (HSIL+). Although AIS and ACC often originate within the endocervical canal, this study demonstrated that all AIS cases were detectable by biopsy alone. Of the 11 ACC cases, only one with a type 3 transformation zone would have been missed without the use of ECC.

The varying detection rates of HSIL+ through ECC across different subgroups underscore the heterogeneity of AGC presentations. The additional detection of HSIL+ relies on two key factors: the risk of HSIL+ and whether the colposcopy comprehensively visualizes the transformation zone where nearly all cervical cancers develop. HPV genotype is a significant risk factor. In women with AGC cytology results, HPV 16/18 infection poses a higher risk compared to other high-risk HPV infections and HPV-negative women (12–14). Numerous studies have indicated that ECC has a higher additional detection rate for HSIL+ in patients positive for HPV 16/18 (6, 15–18). Our findings align with these studies. When AGC is identified cytologically, the cumulative incidence risk of cervical cancer is moderately lower than that of HSIL but considerably higher than that of LSIL (1), thus colposcopy is recommended for all patients regardless of HPV status (19). If colposcopy impression is normal or LSIL, HSIL+ may potentially missed by biopsy alone. Women with AGC-FN cytology have a higher risk of HSIL+, especially AIS+, compared to those with AGC-NOS (20). The type 3 transformation zone poses significant challenges for adequate visualization and sampling during colposcopy, making ECC a valuable adjunctive procedure. Conversely, the absence of missed HSIL+ cases in younger women, those with type 1/2 transformation zones, and those negative for high-risk HPV suggests that ECC may have limited utility in these subgroups.

Our findings concur with previous research indicating a relatively low yield of ECC in detecting additional HSIL+ cases in certain populations. For instance, Solomon et al. (6, 21) reported similar low detection rates of HSIL+ by ECC in women younger than 30 years old. This can be attributed to the lower incidence of cervical cancer in women younger than 30 years old (22) and the predominance of type 1/2 transformation zones in this demographic (23).

In light of the contrasting guidelines regarding ECC, our findings highlight the importance of context and patient-specific factors when considering its use. While the ASCCP recommends ECC for patients with atypical glandular cells to enhance detection rates, the hesitancy of the BSCCP to endorse routine ECC underscores the need for a more nuanced approach. Our results suggest that ECC may be particularly beneficial for women with AGC-FN cytology and specific risk factors like HPV 16/18 infections, yet show limited advantages for younger women and those with type 1/2 transformation zones. This emphasizes the necessity for tailored decision-making in clinical practice, aligning with the evolving discourse on the effectiveness of ECC. This approach could reduce unnecessary procedures in low-risk populations while ensuring high-risk cases are adequately identified and treated.

Several limitations of this study should be acknowledged. First, the retrospective design may introduce selection bias. Second, the sample size, while sufficient for detecting significant differences, may limit the generalization of our findings to broader populations. Additionally, variations in colposcopy and biopsy techniques among practitioners could influence the detection rates of HSIL+.

Future research should focus on prospective studies to validate these findings in larger and more diverse populations. Investigating the molecular and histopathological characteristics of AGC subtypes may provide deeper insights into their malignant potential and guide more precise management strategies. Additionally, exploring the cost-effectiveness of targeted ECC in high-risk subgroups could support evidence-based guidelines for colposcopy referral and follow-up.

## 5 Conclusion

This study aimed to improve the detection rate of HSIL+ in women with AGC while minimizing unnecessary discomfort. We identified high-risk groups warranting ECC: those with a normal/low-grade colposcopic impression, AGC-FN cytology, type 3 transformation zones, and HPV 16/18 infection. Conversely, ECC offers no benefits for women under 30 years old with type 1/2 transformation zones. These findings may reduce missed occult HSIL+ cases and contribute to the evidence base regarding the clinical use of ECC.

## Data Availability

The datasets generated and/or analysed during the current study are not publicly available due personal information protection, patient privacy regulation, and medical institutional data regulatory policies, etc., but are available from the corresponding author on reasonable request.
